# The Relationship of PM Variation with Visibility and Mixing-Layer Height under Hazy/Foggy Conditions in the Multi-Cities of Northeast China

**DOI:** 10.3390/ijerph14050471

**Published:** 2017-04-29

**Authors:** Hujia Zhao, Huizheng Che, Yanjun Ma, Yangfeng Wang, Hongbin Yang, Yuche Liu, Yaqiang Wang, Hong Wang, Xiaoye Zhang

**Affiliations:** 1 State Key Laboratory of Severe Weather (LASW) and Institute of Atmospheric Composition, Chinese Academy of Meteorological Sciences, CMA, Beijing 100081, China; tjzhj4659@sina.com (H.Z.); yaqiang_w@163.com (Y.W.); wangh@camscma.cn (H.W.); xiaoye@camscma.cn (X.Z.); 2 Institute of Atmospheric Environment, China Meteorological Administration, Shenyang 110016, China; mayanjun0917@163.com (Y.M.); wyf_7818@163.com (Y.W.); laoyang105@163.com (H.Y.); liuyuche@sina.cn (Y.L.)

**Keywords:** visibility, PM, MLH, multi-cities, Northeast China

## Abstract

The variations of visibility, PM-mass concentration and mixing-layer height (MLH) in four major urban/industry regions (Shenyang, Anshan, Benxi and Fushun) of central Liaoning in Northeast China are evaluated from 2009 to 2012 to characterize their dynamic effect on air pollution. The annual mean visibilities are about 13.7 ± 7.8, 13.5 ± 6.5, 12.8 ± 6.1 and 11.5 ± 6.8 km in Shenyang, Anshan, Benxi and Fushun, respectively. The pollution load (PM × MLH) shows a weaker vertical diffusion in Anshan, with a higher PM concentration near the surface. High concentrations of fine-mode particles may be partially attributed to the biomass-burning emissions from September in Liaoning Province and surrounding regions in Northeast China as well as the coal burning during the heating period with lower MLH in winter. The visibility on non-hazy fog days is about 2.5–3.0 times higher than that on hazy and foggy days. The fine-particle concentrations of PM_2.5_ and PM_1.0_ on hazy and foggy days are ~1.8–1.9 times and ~1.5 times higher than those on non-hazy foggy days. The MLH declined more severely during fog pollution than in haze pollution. The results of this study can provide useful information to better recognize the effects of vertical pollutant diffusion on air quality in the multi-cities of central Liaoning Province in Northeast China.

## 1. Introduction

The degradation of visibility has been widely studied as one of the key parameters indicative of air quality [1,2,3,4]. Suspended particles, especially fine-mode ones in the atmosphere are the primary factors that impair visibility by scattering and absorbing light [5,6,7,8]. Atmospheric particulate matter (PM) pollution could cause a reduction of visibility [9,10] during heavy pollution periods closely related to the prevailing meteorology [11].

Some studies have shown that visibility degradation has become more serious in urban areas than the rural regions because of the rapid urbanization with a corresponding increase in traffic and higher energy consumption [12,13,14]. The high aerosol loading of anthropogenic origin sources in urban areas contributes to the worse visibility [15] and could influence local and regional air quality [16,17,18]. The atmospheric mixing layer height (MLH) is one of the important meteorological parameters that affect the vertical dispersion of air pollutants and has been studied worldwide [19,20,21,22,23,24].

In China, studies about the MLH and its implication in air pollution have focused more on the main cities [25,26,27,28], but due to the economic growth and urban expansion, visibility degradation has become a new environmental research direction for most urban areas of China [29,30,31,32]. Zhang et al. [33] and Zhang et al. [34] pointed out that the fine particles could be the principal pollutant that causes the worsening visibility in most urban areas in China, so coal combustion and vehicle exhaust emissions could be the primary atmospheric pollution sources causing visibility degradation in China [35]. Furthermore, four urban regions with serious visibility degradation problem have been identified in China, including the Beijing-Tianjin-Hebei Megalopolis (BTH), the Pearl River Delta (PRD), the Yangtze River Delta (YRD), and the Sichuan Basin [36,37,38,39]. In particular, these urban areas have also been revealed as the main hazy regions in China [40,41,42,43,44,45]. Therefore, the urban agglomerations will play a more important role in the research about atmospheric changes from urban to regional scale in China [46,47].

**Figure 1:**
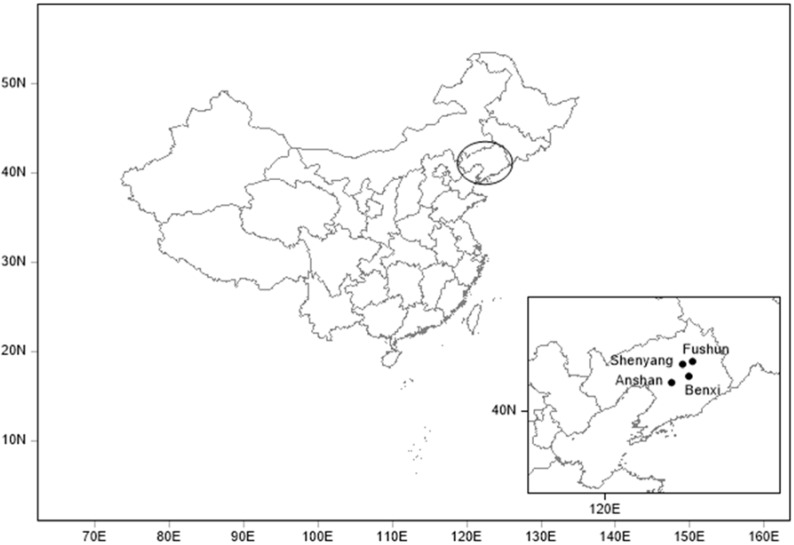
Geographic location of the multi-cities in central Liaoning Province over Northeast China.

However, there are few studies focused on long duration visibility degradation, PM concentration and MLH variation in the urban areas over Northeast China, especially in the “multi-cities” [48]. The multi-cities of central Liaoning Province (40°00′–42°29′ N, 122°10′–125°29′ E) constitute the main development areas in China, with some of the biggest agglomerations of heavy industry and population density in Northeast Asia. The unique geographical environment and economic structure in the multiple cities of central Liaoning have produced many environmental problems, especially atmospheric visibility ones [49,50,51,52]. In this study, we have chosen Shenyang, Anshan, Benxi and Fushun as the representative sites among the multi-cities of central Liaoning (Figure 1), which could feature the typical bad visibility ranges and particulate pollution under the regional boundary layer dynamics found in the typical “multi-city” areas.

The long-term record of daily visibility, PM (PM_10_, PM_2.5_ and PM_1.0_) mass concentrations data and MLH over a period of nearly four years (from June 2009 to December 2012) in the multi-cities of central Liaoning in Northeast China are characterized in this study. The potential relationships between visibility, PM and MLH were also investigated, along with relative humidity (RH) and wind direction, respectively. The primary objectives of this paper are to: (1) present the characteristics of long-term observations of visibility, PM concentration and MLH in those multi-cities in central Liaoning in Northeast China; (2) better understand the effect of MLH on air pollution correlated with meteorological parameters in the multi-cities of central Liaoning. This research examines the regional air quality on the typical “multi-cities” in Northeast China, which should be helpful to provide information to support possible strategies of visibility improvement and particulate matter reduction in other Chinese metropolitan areas.

## 2. Materials and Methods

In this paper, four sites in the multi-cities of central Liaoning over Northeast China were selected: Shenyang (41.77° N, 123.50° E, 60.0 m) is the capital of Liaoning Province, as well as the political, economic and cultural center in Northeast China which could represent the metropolitan areas. Anshan (41.08° N, 123.00° E, 23.0 m), Benxi (41.32° N, 123.78° E, 183.0 m) and Fushun (41.88° N, 123.95° E, 80.0 m) are three other important sites in central Liaoning Province with different geographical features, which reflect the urban/industrial aerosol characteristics in Northeast China. The multi-cities of central Liaoning are the important economic development region in Northeast China, which is affected by both industrial emissions and residential activities. The air quality there could be linked to the local industry development as well as the transportation in the nearby regions of the multi-cities in central Liaoning with an obvious effect on the regional pollution in Northeast China.

A FD12 visibility automatic observation instrument (Vaisala, Helsinki, Finland) and a GRIMM 180 particle detection instrument (GRIMM Aerosol Technik, Ainring, Germany) were used to obtain visibility and PM (PM_10_, PM_2.5_ and PM_1.0_) mass concentrations at the four sites since June 2009. The measuring time of the FD12 is 15 s with an accuracy of ±10% between 0.01 km–10 km and ±20% between 10 km–50 km, and the measuring range is 10–50,000 m. The measuring time of the GRIMM 180 is 1–60 min, with an accuracy of ±2% and the measuring range is 1–1500 µg/m^3^. The daily and monthly values of visibility and PM-mass concentration were calculated using statistical analysis using the 10-min average visibility measurements and 5-min average mass concentration measurements to characterize their properties. The observation days of visibility and PM (PM_10_, PM_2.5_ and PM_1.0_) at Shenyang, Anshan, Benxi and Fushun were 1232, 1295, 1044, 426 and 1220, 1272, 1188, 1174, respectively. The fewer visibility data in Fushun were not as good as the date sets from the other three sites due to instrument problems. In addition, the daily meteorological data, including relative humidity (RH), wind speed and direction, were also collected from June 2009 to December 2012. The daily MLH was obtained as average of hourly MLH, which was calculated based on the total cloud cover, low cloud cover and wind speed according to the Technical Guidelines for Environmental Impact Assessment of China [53]. In view of the dynamics and thermodynamics, the formulae for the evaluation of MLH are different depending on the stability, and are divided into classes (A, B, C, D, E or F). When the atmospheric stability grade is A, B, C and D:

*h* = *a*_s_*U*_10_/*f* (1)

where *h*: MLH (m).

When the atmospheric stability grade is E and F:

(2) $$h=b_{s}\sqrt{U_{10}}/f$$

*f* = 2*Ω*sin *φ* (3)

where *U*_10_: the wind speed at the height of 10 m (m/s); *a*_s_, *b*_s_: are mixing layer coefficients in China (*a*_s_ was about 0.037, 0.06, 0.041 and 0.019 when the atmospheric stability grade is A, B, C and D; *b*_s_ was about 1.66 and 0.70 when the atmospheric stability grade is for E and F in this paper); *f*: Earth rotation parameter; *Ω*: angular velocity (7.29 × 10^−5^ rad/s); *φ*: geographic latitude (°).

## 3. Results and Discussion

### 3.1. Annual Average of Visibility, PM Mass Concentration and MLH in the Four Sites

Table 1 lists the multi-annual values of visibility, PM-mass concentration (PM_10_, PM_2.5_ and PM_1.0_) and MLH from June 2009 to December 2012 in the multi-cities of central Liaoning.

The multi-annual visibility was about 13.7 ± 7.8, 13.5 ± 6.5, 12.8 ± 6.1 and 11.5 ± 6.8 km during the 4-year period in Shenyang, Anshan, Benxi and Fushun, respectively. The multi-annual mean visibility in these four sites was much lower than the national averaged value of ~26.00 km according to Che et al. [47], which suggests the poor atmospheric quality over the multi-cities of central Liaoning in Northeast China. The multi-annual PM_2.5_-mass concentrations were about 49.1 ± 27.3, 58.8 ± 36.3, 56.4 ± 33.1 and 43.9 ± 28.9 μg/m^3^ in Shenyang, Anshan, Benxi and Fushun, respectively, which all exceeded the annual limit of China’s national ambient air-quality standards (35 µg/m^3^; GB3095-2012 [54]). In addition, the annual concentration of PM_10_ in Anshan was 102.0 ± 63.3 µg/m^3^, which was almost 1.2–1.4 times higher than that at the other three sites; the results indicate an obvious local pollution of coarse-mode particles in this urban/industrial area. The multi-annual MLH was about 535.8 ± 207.0, 517.4 ± 212.7, 457.6 ± 195.9 and 484.1 ± 191.0 m in Shenyang, Anshan, Benxi and Fushun, respectively. The MLH is a parameter that indicates the pollution dilution effects which means that Shenyang with a higher MLH than the other three sites has better air-quality conditions.

Furthermore, the pollution load (PM × MLH) has been considered in this study in order to exclude the influence of MLH on PM mass concentration, according to He et al. [55] and Li et al. [56]. According to the list of Table 1, the near-surface particulate matter concentration in Anshan was the highest among the four sites, while the pollution load (PM × MLH) in the mixing layer was not extremely higher. However, the particulate-matter concentration in the near-surface is lower in Shenyang, while the pollution load (PM × MLH) was higher in the mixing layer among the four sites. These results show that the weaker vertical diffusion may cause high PM concentrations in the near-surface in Anshan, while in Shenyang, the highest pollution load (PM × MLH) may be due to the contribution of pollutant transportation and the local emission sources [50].

### 3.2. Monthly and Seasonal Variations of Visibility, MLH and Meteorology in the Four Sites

**Figure 2:**
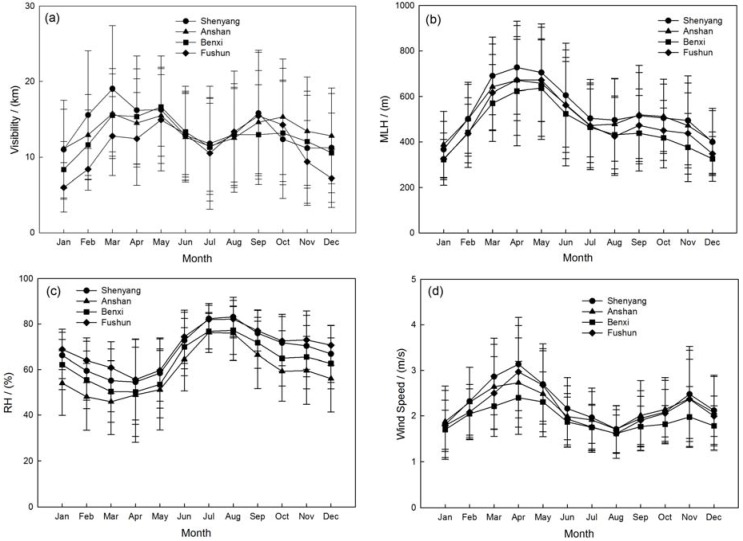
Monthly mean variations of (**a**) visibility; (**b**) MLH; (**c**) RH and (**d**) Wind speed with the error bars represent the standard deviations in the multi-cities of central Liaoning in the period June 2009 to December 2012.

As seen in Figure 2, the monthly visibility is higher in March and September and lower in July and January in the four multi-cities. The maximum visibility of about 19.1 ± 8.4 km occurred in March over Shenyang and the minimum in January over Fushun, with an average value of about 6.0 ± 3.3 km. There is a similar pattern between visibility and MLH. The monthly mean MLH shows a peak value in April (623–726 m) and falls to a lower value in August (424–496 m). Then the MLH increases to a secondary peak in September (438–519 m) and decreases again in January (322–387 m). The RH is lower (less than 60%) in March–April–May, while higher in June–July–August (more than 80%). Compared with the higher temperature, the high RH has enhanced the photochemical transformation of secondary aerosols, favoring higher concentration of fine-mode particles [57]. The monthly mean wind speed is about 2.5 m/s in April and reduces to 2.0 m/s in August, then increases to the high speed of ~3.0 m/s in November, then continues to reduce to 2.0 m/s in January.

**Figure 3:**
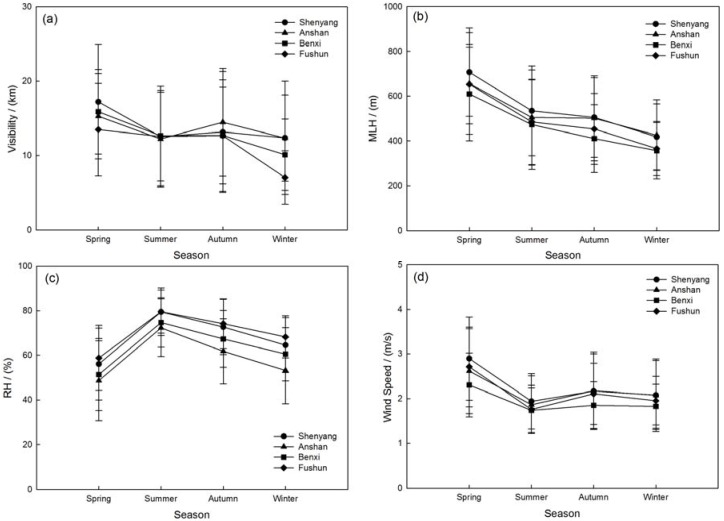
Seasonal mean variations of (**a**) visibility; (**b**) MLH; (**c**) RH and (**d**) Wind speed with the error bars represent the standard deviations in the multi-cities of central Liaoning in 2012.

The seasonal visibility is obviously worse in winter accompanied with the lower mixing layer height of about 400–500 m in the four sites. The maximum visibility occurred in spring with the larger wind speed about 2–3 m/s as Figure 3d illustrates. The higher RH (~80%) and lower wind speed (~1.8 m/s) in summer may be the main factors causing lower visibility. The variation of MLH may be related to the seasonal radiation flux during the year to affect visibility by vertical pollutants diffusion as Figure 3 describes [58,59]. The patterns of wind speed reveal that a large number of aerosols have being carried into the atmosphere due to strong winds in spring. The significantly lower wind speeds in summer possibly cause a high PM concentration accumulation, which induces a reduction in visibility. The increasing wind speed in autumn accelerates the aerosol concentration from biomass burning by regional transportation. The slightly weaker winds in the winter limit the dispersion of pollutants in the cold season.

**Figure 4:**
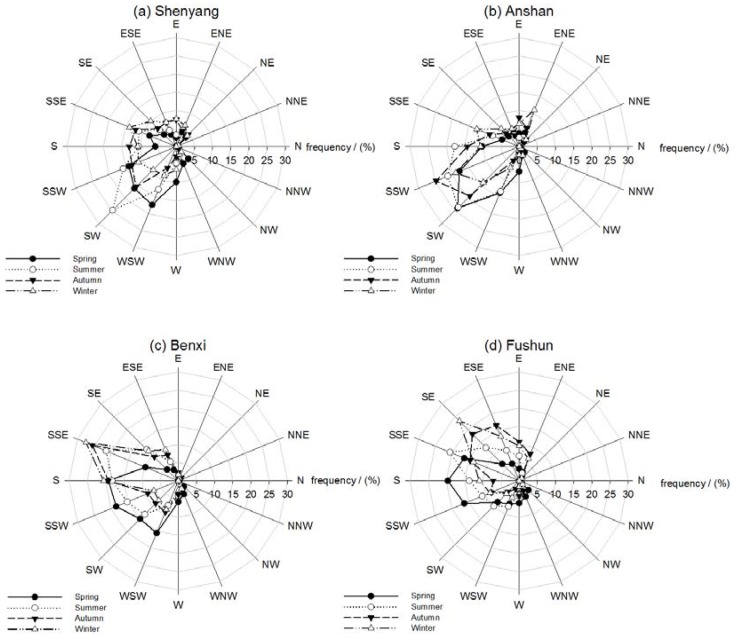
Wind direction roses in (**a**) Shenyang; (**b**) Anshan; (**c**) Benxi and (**d**) Fushun in the multi-cities of central Liaoning in the period June 2009 to December 2012.

The prevailing winds in different seasons during the observation period over the four sites are also shown in Figure 4. The seasonal wind patterns could contribute to the transport of pollutants influencing the PM-mass concentrations. In spring (March–April–May), the prevailing wind flow is mainly from the south or southwest with a poor atmospheric stability, which easily forms dusty conditions. In summer, the prevailing wind is mainly from the south or southeast, which could provide regional transport of water vapor from southern regions that favor the formation of foggy weather. Affected by the high pressure caused by Changbai Mountain, the prevailing winds are mainly from the southeast in the autumn and winter, when cold air falls from the mountain and causes a strong radiative cooling on the ground to form an inversion layer. This climate mechanism leads to an accumulation of contamination close to the ground.

### 3.3. Monthly Variations of Coarse and Fine Mode Particles Mass Concentration in the Four Sites

**Figure 5:**
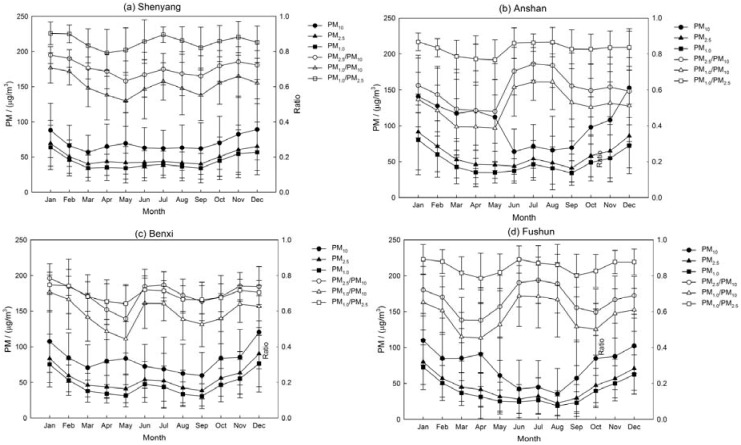
Monthly variations of PM-mass concentration and PM Ratio with the error bars represent the standard deviations in (**a**) Shenyang; (**b**) Anshan; (**c**) Benxi and (**d**) Fushun in the multi-cities of central Liaoning in the period June 2009 to December 2012.

In this section, the significantly different characteristics of the coarse- and fine-mode particle mass concentration over the four sites are described in Figure 5. The concentration of coarse-mode particles shows an obvious surge in March–April–May, which represents the large number of coarse-mode aerosols produced by dust storms; wind erosion in the spring results in high aerosol loading [50]. However, the corresponding visibility in this period is not decreased, which denotes that the coarse particles are not the main cause affecting visibility in the spring. There were two peaks in the fine-mode particles concentration distribution which is usually observed in June–July–August and October-November–December–January the next year. The longer and stronger solar irradiation during summer can favor photochemical formation of secondary aerosol particles that lead to higher fine-particle levels in this period [60,61]. However, more precipitation could reduce the concentration of aerosols to a smaller peak value.

Especially, it is interesting to note that the fine-particle concentration increased somewhat from September to November and maintains a steady level lasting the whole winter even into January of the next year. High concentrations of fine-mode particles may be partially attributed to the biomass-burning emissions from September, which is the most active month in Liaoning Province and surrounding regions in Northeast China [62,63]. In addition, the fine mode particles increased in the winter as a result of a combination of increased emissions from heating sources and low MLH as Figure 2b shows [64,65]. Residential heating may be one of the typical pollution sources in the multi-cities that leads to the highest BC concentration occurring in winter [46,47,66,67].

Figure 5 also shows that the ratios of PM_2.5_/PM_10_ and PM_1.0_/PM_10_ obviously increase in summer and winter, while in spring the ratio decreases significantly. The change of PM ratio is small in Shenyang while it is remarkable in the other three sites. The steady higher level of PM ratio in Shenyang highlights the contribution of anthropogenic pollutants due to the soaring urbanization from being the capital of Liaoning Province. In general, dust events, combustion activities as well as secondary aerosols are the three major sources of PM in the multi-cities of Liaoning Province in Northeast China.

### 3.4. Relationship between Visibility, PM-Mass Concentration and MLH

**Figure 6:**
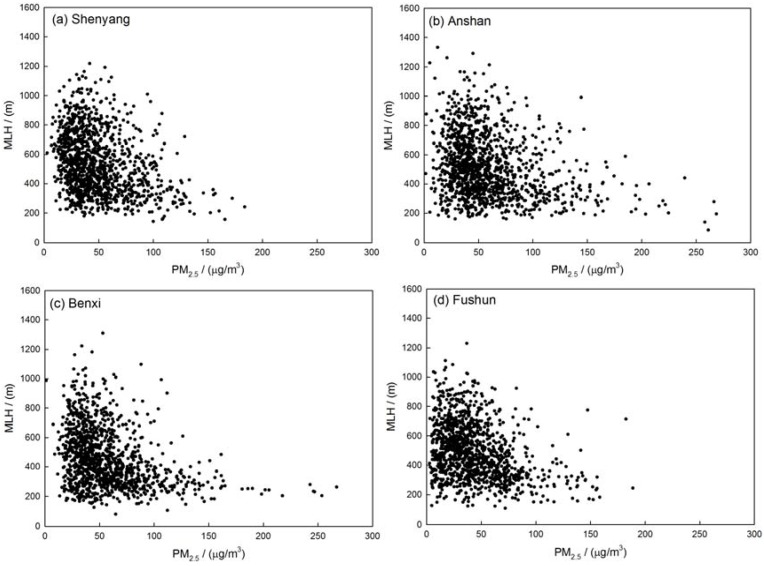
Scatter plot of PM_2.5_-mass concentration and MLH in (**a**) Shenyang; (**b**) Anshan; (**c**) Benxi and (**d**) Fushun in the multi-cities of central Liaoning in the period June 2009 to December 2012.

**Figure 7:**
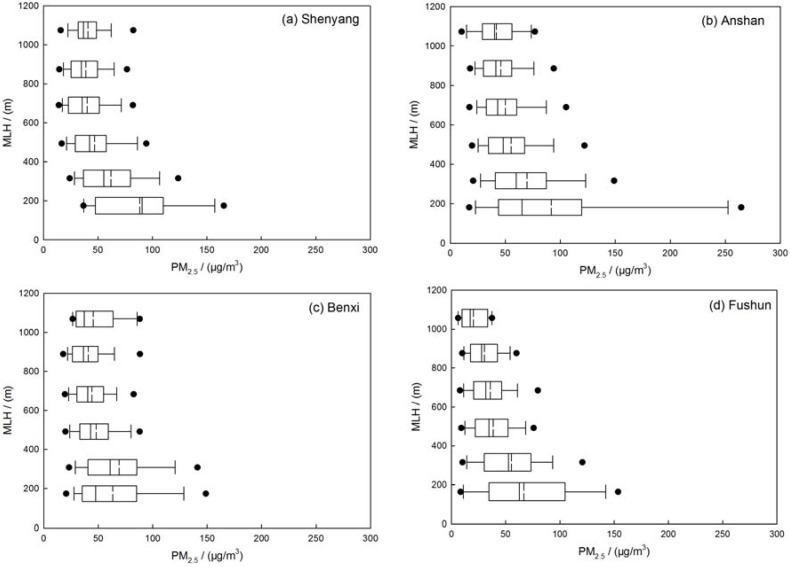
Distribution of average PM_2.5_ mass concentration and the 5th and 95th percentile box plots in (**a**) Shenyang; (**b**) Anshan; (**c**) Benxi and (**d**) Fushun in the multi-cities of central Liaoning in the period June 2009 to December 2012.

**Figure 8:**
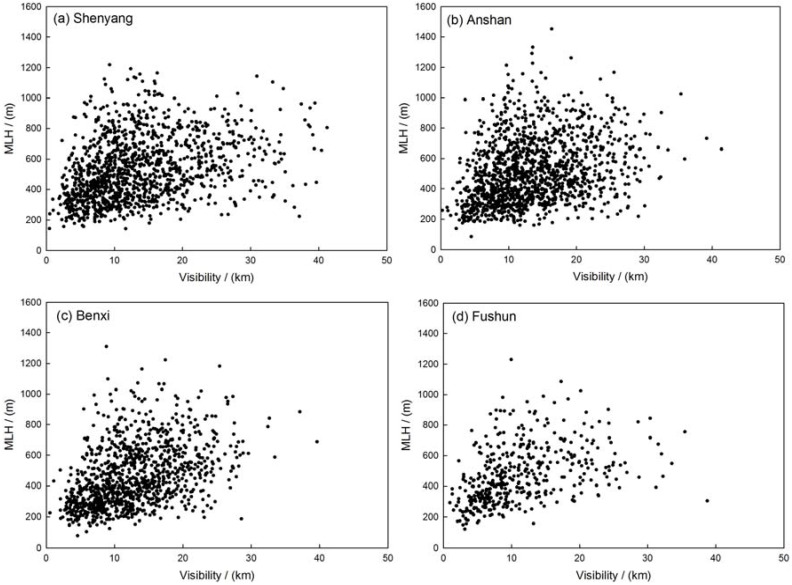
Scatter plot of visibility and MLH in (**a**) Shenyang; (**b**) Anshan; (**c**) Benxi and (**d**) Fushun in the multi-cities of central Liaoning in the period June 2009 to December 2012.

**Figure 9:**
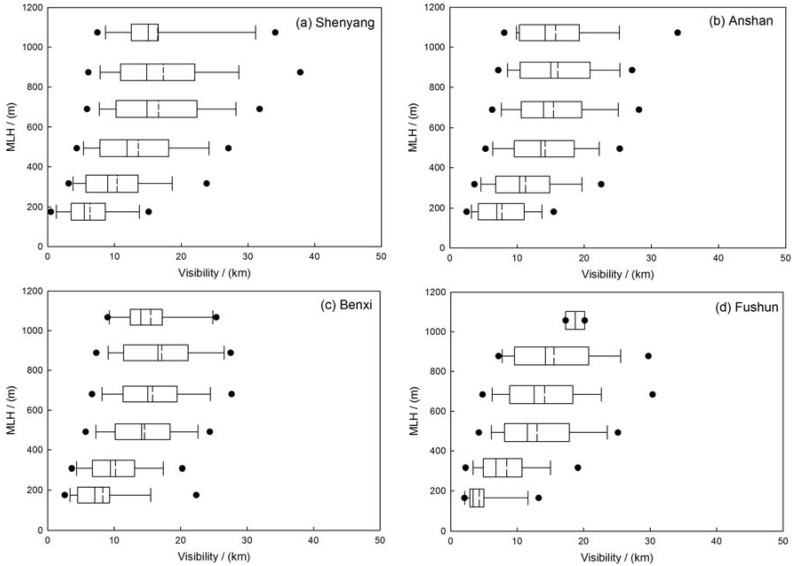
Distribution of average visibility and the 5th and 95th percentile box plots in (**a**) Shenyang; (**b**) Anshan; (**c**) Benxi and (**d**) Fushun in the multi-cities of central Liaoning in the period June 2009 to December 2012.

The correlation between visibility, coarse- and fine-mode particle mass concentrations and MLH has been established to simply describe the vertical transport of particles in the four sites. The correlation coefficient of MLH and PM_2.5_ are about −0.31, −0.27, −0.33, −0.33, or said otherwise, the correlation coefficients between MLH and PM_10_ are about −0.24, −0.05, −0.20, −0.14 in Shenyang, Anshan, Benxi and Fushun, respectively. The results show that the mixing layer height may have more effect on fine mode particles than coarse mode particles (Table 2). Figure 6 indicates that the PM_2.5_-mass concentration increased exponentially with decreasing MLH. The distribution of the PM_2.5_ concentrations along with different MLH has been represented in Figure 7. When mixing layer height increased, the concentration of PM_2.5_ decreased. The average concentration of PM_2.5_ decreased to about 50.0 µg/m^3^ with MLH rising from ground to about 500 m. When the MLH reached more than 500 m and the PM_2.5_ mass concentration did not vary obviously with the increasing height of MLH. The correlation between visibility and MLH is plotted in Figure 8. The correlation coefficients of the visibility and MLH are 0.32, 0.28, 0.42 and 0.40 in Shenyang, Anshan, Benxi and Fushun, respectively, which indicates that higher MLH has a good influence on atmospheric visibility (Table 2). The distribution of visibility with MLH has been shown in Figure 9. The visibility becomes better with the increasing height of MLH and when the MLH increased to about 700 m, the visibility varies slowly. Moreover, the relationship between visibility and MLH under different RH ranges is also considered but the results show that the relationship between visibility and MLH is not improved as RH increases compared to the studies of Tang et al. [27]. Tang et al. [27] indicated that high RH may favor local contribution of humidity-related physicochemical processing in heavy pollution which needs the vertical transportation of MLH to get better visibility. In this paper, RH does not have the same significant impact on aerosol physicochemical processing to enhance the correlation between visibility and MLH which a fact that needs further investigation.

**Figure 10:**
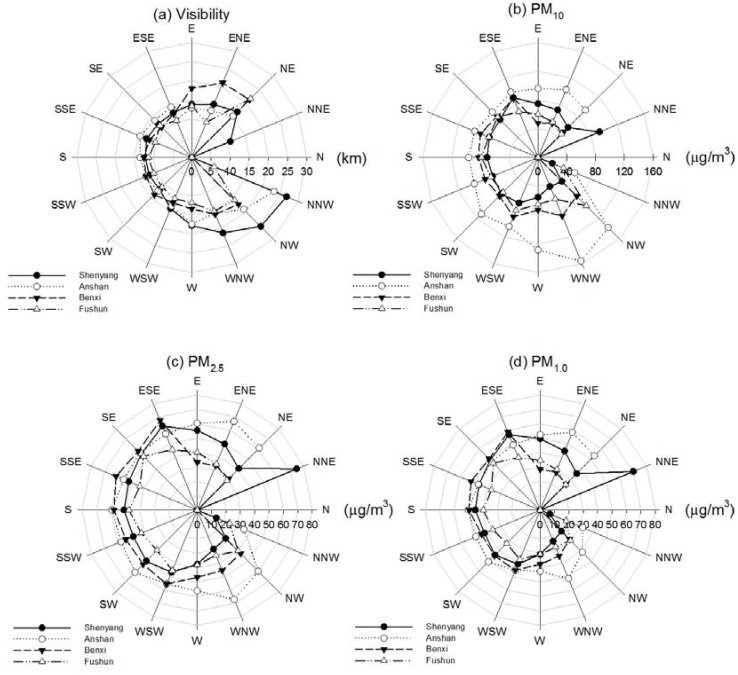
Roses of (**a**) visibility; (**b**) PM_10_; (**c**) PM_2.5_ and (**d**) PM_1.0_ for various wind directions in the multi-cities of central Liaoning in the period June 2009 to December 2012.

Co-variation between visibility, PM concentration and wind direction in the multi-cities is also shown in Figure 10 to describe the horizontal variation in air pollution. In Shenyang, the wind flow from the North-West has the larger visibility, about 25 km, compared with the lower level of PM concentration. In Anshan, the wind flow from the North-North-West has the larger visibility, about 23 km, compared with the lower level of PM concentration. Unlikely in Shenyang and Anshan, the wind directions in North-East have a larger visibility of about 21 km compared with the lower PM concentration in Benxi and Fushun. On the contrary, the wind flow from the North-North-East has the higher PM concentration with lower visibility about 10 km in Shenyang. In the other three sites, the wind flow from the South-West is associated with the largest PM concentration. Therefore, the airflow from North of Liaoning Province could carry the clear air to the multi-cities while the airflow from South-West may convey polluted air from inland China.

### 3.5. Variations in Visibility and PM during Pollution Episodes

**Figure 11:**
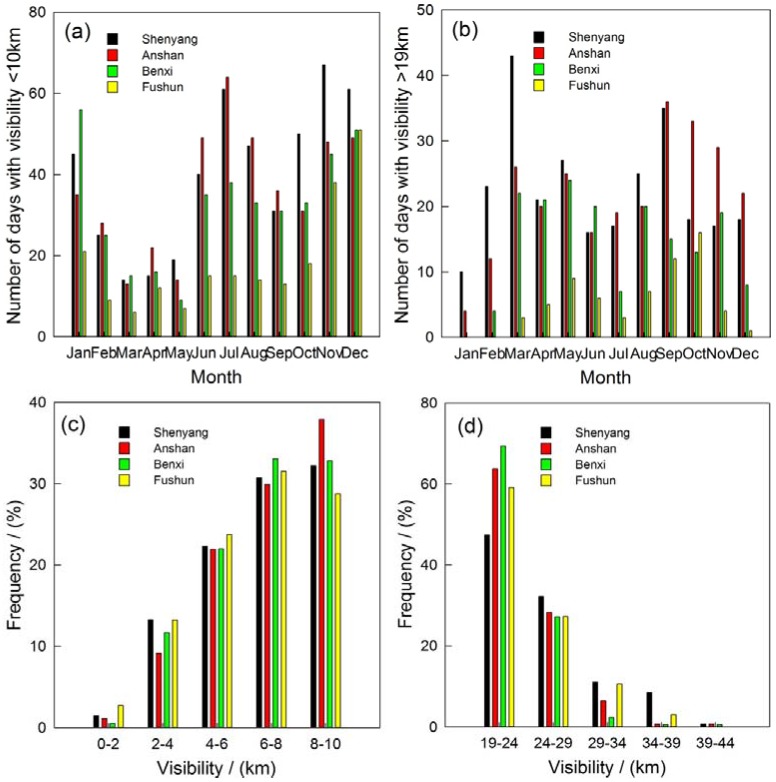
Numbers of days for (**a**) visibility <10 km and (**b**) visibility >19 km and frequency of occurrence for (**c**) visibility <10 km and (**d**) visibility >19 km in the multi-cities of central Liaoning in the period June 2009 to December 2012.

The days and distribution of visibility >19 km and <10 km in the multi-cities of central Liaoning is shown in Figure 11, classified according to Gomez and Smith [68], which has an inverse correlation in contrast to the MLH.

The days of visibility <10 km occur from June to January and February in the next year, while the days of visibility >19 km are more frequent in March-April-May. The distribution of low visibility between 8.0 km and 10.0 km has the most frequent occurrence for about 28.8–37.9%. The low visibility ranges in 6.0–8.0 km account for 29.9–33.1% of the total occurrence. The low visibility of less than 1 km is extremely scarce in the four sites. The good visibility ranges in 19–24 km and 24–29 km account for 47.4–69.4% and 27.2–32.2%, respectively.

In this study, a haze episode is defined as RH < 90% and visibility ≤10 km [69]. Non-haze period is the one with RH < 90% and visibility >10 km. Fog episode is under the conditions of RH ≥ 90% and visibility ≤10 km [70]. The GRIMM 180 particle instrument was used to obtain PM mass concentration in this paper which may have some influenced the final measurement uncertainty by relative humidity according to Dinoi et al. [71], but the PM data still can be used to recognize the fog-haze pollution to a certain degree.

The variations of visibility, PM-mass concentration and MLH under different meteorological conditions during haze, fog, non-hazy/foggy days are shown in Table 3. The visibility on the non- hazy/foggy days is about 2.5 and 3.0 times higher than that on hazy and foggy days in the four multi-cities. The fine-particle concentrations of PM_2.5_ and PM_1.0_ on the hazy days are ~1.8–1.9 times higher than that on non-hazy/foggy days, while on foggy days, the concentrations of PM_2.5_ and PM_1.0_ are ~1.5 times higher than that on non-hazy/foggy days in the four multi-cities. For Anshan, a lower PM_10_ mass concentration during foggy days than that observed in non-hazy/foggy days was observed, which may be due to the wet deposition of coarse-particle accompanied with higher RH in foggy days.

The RH during hazy and foggy days are 74.4 ± 10.3%, 66.8 ± 13.8%, 70.5 ± 11.2%, 73.2 ± 10.0% and 94.1 ± 3.1%, 94.2 ± 2.6%, 94.2 ± 2.9%, 91.2 ± 1.0% for Shenyang, Anshan, Benxi and Fushun, respectively, which is much higher than the values on non-hazy days. The average wind speed during hazy and foggy days is 2.0 ± 0.8 m/s, 2.0 ± 0.9 m/s, 1.8 ± 0.6 m/s, 2.0 ± 0.7 m/s and 1.8 ± 0.6 m/s, 1.9 ± 0.7 m/s, 1.4 ± 0.4 m/s, 1.5 ± 0.4 m/s for Shenyang, Anshan, Benxi and Fushun, respectively, as compared to non-hazy days.

**Figure 12:**
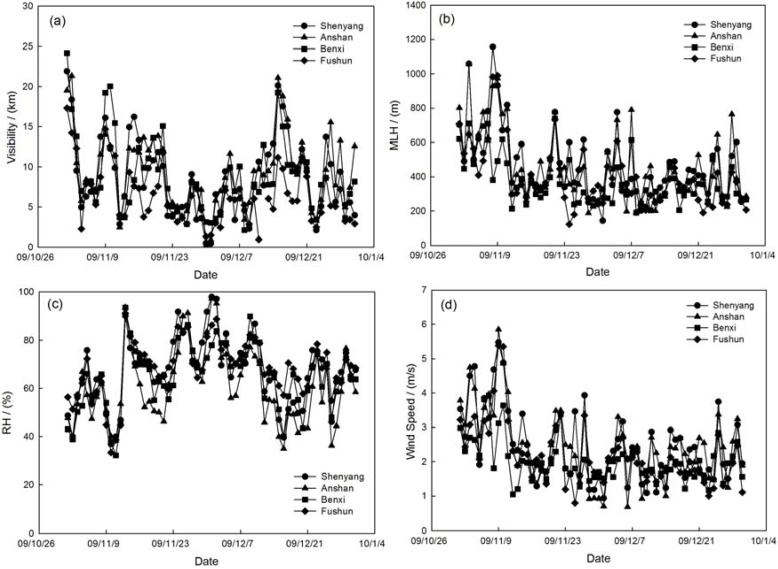
The variation of (**a**) visibility; (**b**) MLH; (**c**) RH and (**d**) wind speed during the fog episode of 29 November to 1 December 2009 in the multi-cities of central Liaoning.

**Figure 13:**
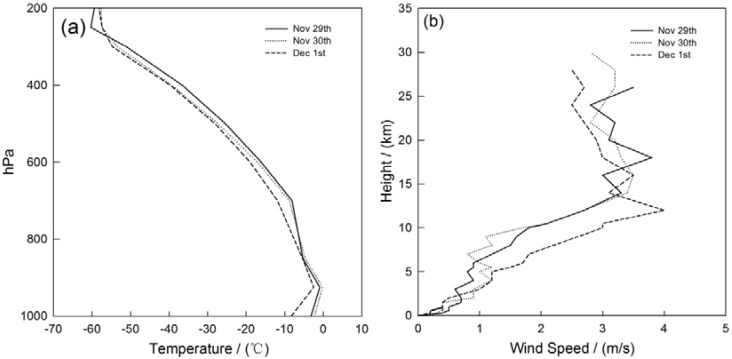
The vertical variation of (**a**) temperature and (**b**) wind speed during the fog episode of 29 November to 1 December 2009 in the multi-cities of central Liaoning.

The MLH is an important meteorological factor to analyze the dynamical effects air pollution. The MLH during haze and fog episodes is about 467.7 ± 187.6 m, 449.3 ± 202.5 m, 376.3 ± 171.5 m, 410.1 ± 175.4 m and 379.5 ± 174.3 m, 427.0 ± 161.2 m, 285.9 ± 104.8 m and 347.2 ± 117.6 m at Shenyang, Anshan, Benxi and Fushun, respectively. The MLH on the non-hazy/foggy days is about 1.2 and 1.5 times higher than that on hazy and foggy days. These results compared with non-haze-fog days dictate that the MLH during fog pollution shows a more declining trend than haze pollution, which indicates the relatively large impact of dynamic effects on fog pollution in the multi-cities of central Liaoning. Through the analysis of a fog episode on 29 November to 1 December 2009 over the multi-cities of central Liaoning in Northeast China, the horizontal and vertical meteorological parameters are shown in Figure 12 and Figure 13. As the thermal/dynamic parameters describe, the MLH is lower by almost 200 m compared with the stable weather conditions of temperature inversion and low wind speed in the near-surface during the fog pollution by significant visibility deterioration.

## 4. Conclusions

In this study, the long-term visibility, PM-mass concentration and mixing layer height were investigated from 2009 to 2012 in the multi-cities of central Liaoning in Northeast China to represent the effects of horizontal and vertical meteorology on air pollution in these growing urban/industrial areas. The following conclusions may be reached:

(1) The lower annual mean visibility in the multi-cities of central Liaoning suggests a poor atmospheric quality in Northeast China. The pollution load (PM × MLH) shows the higher PM concentration in the near-surface with a weaker vertical diffusion in Anshan. The highest pollution load (PM × MLH) in Shenyang may be due to the contributions of pollutant transportation and local emission sources.
(2) The monthly variation of MLH may be related to the seasonal radiation flux during the year that affects visibility by vertical pollutant diffusion. The increased fine-particle concentration from September to November and even into January in the next year was partially attributed to the biomass-burning emissions and heating sources under lower MLH in winter.
(3) The MLH may have more effect on fine mode particles than coarse mode particles.
(4) The MLH on the non-hazy/foggy days was about 1.2 and 1.5 times higher than that on hazy and foggy days. This indicates that the MLH during fog events shows a more declining trend than during haze events, a fact that indicates the relatively large impact of dynamic effects on fog pollution in the multi-cities of central Liaoning.

However, further studies are still needed to take into account more boundary layer data and their impacts on air pollution in Northeast China.

## Figures and Tables

**Table 1:** Annual averages of visibility, PM-mass concentration and MLH (mixing-layer height) in the multi-cities of central Liaoning.

**Sites**	**Visibility (km)**	**PM_10_ (μg/m^3^)**	**PM_2.5_ (μg/m^3^)**	**PM_1.0_ (μg/m^3^)**
Shenyang	13.7 ± 7.8	69.8 ± 37.6	49.1 ± 27.3	43.1 ± 26.2
Anshan	13.5 ± 6.5	102.0 ± 63.3	58.8 ± 36.3	49.2 ± 31.8
Benxi	12.8 ± 6.1	81.8 ± 45.3	56.4 ± 33.1	47.5 ± 29.2
Fushun	11.5 ± 6.8	71.8 ± 55.2	43.9 ± 28.9	37.2 ± 25.5
**Sites**	**PM_10_ × MLH (mg/m^2^)**	**PM_2.5_ × MLH (mg/m^2^)**	**PM_1.0_ × MLH (mg/m^2^)**	**MLH (m)**
Shenyang	35.5 ± 21.3	24.5 ± 14.0	21.3 ± 12.9	535.8 ± 207.0
Anshan	51.7 ± 40.5	28.1 ± 17.2	23.3 ± 14.7	517.4 ± 212.7
Benxi	35.8 ± 23.5	23.8 ± 13.2	19.8 ± 11.5	457.6 ± 195.9
Fushun	33.7 ± 30.9	19.7 ± 12.7	16.5 ± 10.4	484.1 ± 191.0

**Table 2:** Correlation coefficient between PM-mass concentration, visibility and MLH in the four sites.

Site	PM_10_ vs. MLH	PM_2.5_ vs. MLH	PM_1.0_ vs. MLH	VIS vs. MLH
Shenyang	−0.24	−0.31	−0.33	0.32
Anshan	−0.05	−0.27	−0.30	0.28
Benxi	−0.20	−0.33	−0.35	0.42
Fushun	−0.14	−0.33	−0.36	0.40

**Table 3:** The average visibility, PM-mass concentration, PM ratio and meteorology during the hazy, foggy and non hazy/foggy episodes in the multi-cities of central Liaoning in the period June 2009 to December 2012.

**Site**	**Shenyang**	**Anshan**
VIS (km)/RH (%)/WS (m/s)/MLH (m)		
Haze	6.7 ± 2.1/74.4 ± 10.3/2.0 ± 0.8/467.7 ± 187.6	7.0 ± 2.1/66.8 ± 13.8/2.0 ± 0.9/449.3 ± 202.5
Fog	6.1 ± 2.6/94.1 ± 3.1/1.8 ± 0.6/379.5 ± 174.3	6.3 ± 2.3/94.2 ± 2.6/1.9 ± 0.7/427.0 ± 161.2
Non hazy/foggy	18.1 ± 6.8/63.3 ± 15.0/2.4 ± 0.9/585.5 ± 203.5	16.9 ± 5.3/54.7 ± 16.1/2.3 ± 0.9/555.0 ± 210.0
PM_10_/PM_2.5_/PM_1.0_ (μg/m^3^)		
Haze	94.7 ± 38.9/70.8 ± 30.1/64.0 ± 28.7	126.9 ± 72.5/87.5 ± 44.3/75.2 ± 38.8
Fog	82.6 ± 66.2/53.5 ± 27.8/48.6 ± 26.2	85.1 ± 64.0/71.4 ± 52.1/61.7 ± 40.2
Non haze-fog	57.5 ± 26.4/38.4 ± 17.4/32.6 ± 16.8	92.2 ± 54.8/45.3 ± 19.7/36.9 ± 17.0
**Site**	**Benxi**	**Fushun**
VIS (km)/RH (%)/WS (m/s)/MLH (m)		
Haze	6.9 ± 2.1/70.5 ± 11.2/1.8 ± 0.6/376.3 ± 171.5	6.4 ± 2.2/73.2 ± 10.0/2.0 ± 0.7/410.1 ± 175.4
Fog	6.1 ± 1.9/94.2 ± 2.9/1.4 ± 0.4/285.9 ± 104.8	6.0 ± 2.9/91.2 ± 1.0/1.5 ± 0.4/347.2 ± 117.6
Non hazy/foggy	16.4 ± 4.9/60.4 ± 14.7/2.0 ± 0.6/499.0 ± 193.6	16.9 ± 5.8/68.6 ± 12.8/2.2 ± 0.8/511.0 ± 189.0
PM_10_/PM_2.5_/PM_1.0_ (μg/m^3^)		
Haze	106.5 ± 47.2/80.0 ± 36.2/68.7 ± 32.6	93.2 ± 57.8/64.2 ± 32.3/55.2 ± 28.9
Fog	100.0 ± 79.2/72.5 ± 49.9/58.6 ± 38.0	81.5 ± 105.1/51.4 ± 48.2/44.4 ± 41.0
Non haze-fog	71.7 ± 38.2/46.4 ± 24.4/38.5 ± 21.3	68.1 ± 50.6/40.3 ± 25.9/34.1 ± 23.0
